# A distinctive colour associated with high iodine content in malignant pleural effusion from metastatic papillary thyroid cancer: a case report

**DOI:** 10.1186/1752-1947-7-147

**Published:** 2013-05-31

**Authors:** Andrew Rosenstengel, Ee Mun Lim, Michael Millward, YC Gary Lee

**Affiliations:** 1 Department of Respiratory Medicine, Sir Charles Gairdner Hospital, Hospital Avenue, Nedlands, Perth, WA, 6009, Australia; 2 Department of Clinical Biochemistry, PathWest Laboratory Medicine WA, QEII Medical Centre, Hospital Avenue, Nedlands, Perth, WA, 6009, Australia; 3 Department of Medical Oncology, Sir Charles Gairdner Hospital, Hospital Avenue, Nedlands, Perth, WA, 6009, Australia; 4 School of Medicine and Pharmacology, University of Western Australia, 35 Stirling Highway, Crawley, WA, 6009, Australia; 5 Centre for Asthma, Allergy & Respiratory Research, University of Western Australia, 35 Stirling Highway, Crawley, WA, 6009, Australia

**Keywords:** Pleural effusion, Iodine, Thyroglobulin, Diagnosis, Thyroid, Carcinoma, Colour

## Abstract

**Introduction:**

Pleural effusions are a common clinical problem and affect about one million people in the United States and United Kingdom each year. Over 60 causes of pleural effusion have been identified; establishing the definitive aetiology can be difficult, and often requires invasive procedures. Guidelines state that macroscopic examination of the fluid should be the first step in determining the aetiology of a pleural effusion. Papillary thyroid carcinoma is an uncommon cause of malignant pleural effusion, with only 10 cases reported in the literature, their physical characteristics and composition having been rarely described. We describe for the first time a distinctive brown colour of the malignant effusion (despite centrifugation) from a rare case of metastatic papillary thyroid cancer to the pleura, associated with a high pleural fluid iodine content. Such a characteristic may be useful in expediting diagnosis of a malignant pleural effusion in the appropriate clinical context.

**Case presentation:**

We present the case of a 71-year-old Caucasian man with metastatic papillary thyroid cancer; a large, long-standing, right-sided pleural effusion and a 83-fold higher pleural thyroglobulin level compared to corresponding serum, supporting this malignancy as the cause of the patient’s effusion. The pleural fluid had a distinctive pigmentation similar to iodine-containing antiseptic preparations. Biopsy during medical thoracoscopy confirmed metastatic papillary thyroid carcinoma. Analysis of pleural fluid showed a pleural thyroglobulin level over 80 times that of serum levels (29,000μg/L versus 350ug/L). Pleural fluid iodine content was 23,000ug/L and may account for the fluid’s distinctive pigment, as iodine is an essential component in thyroglobulin and thyroid hormone synthesis.

**Conclusions:**

Pleural fluid pigmentation may aid diagnosis in the appropriate clinical setting. A distinctive iodine-like brown colour of pleural fluid may represent elevated iodine content and should raise consideration of metastatic thyroid cancer as a cause for a pleural effusion.

## Introduction

Pleural effusion is common in clinical practice and can be caused by over 60 pulmonary or systemic disorders. Clinical guidelines [1] and conventional texts [2] recommend that inspection of the fluid can provide useful information: pus indicates empyema, milky fluids are likely to be chylous, blood suggests a haemothorax and food particles indicate oesophageal leaks or fistulae from the gastrointestinal tract. To the best of our knowledge, a dark brown pleural effusion has not been described.

Malignant pleural effusions are a common clinical problem, and a wide variety of malignancies can metastasise to the pleura. Papillary thyroid carcinoma is an uncommon cause of malignant pleural effusion, with only 10 cases in total reported in the literature [3-9], their physical characteristics rarely documented and their composition (for example thyroglobulin levels) seldom described. Iodine is an essential element in the pathway of hormone synthesis in the thyroid gland. To the best of our knowledge, pleural fluid iodine content has not previously been assayed.

We report for the first time a distinctive dark brown pigmentation of the pleural fluid from metastatic malignant papillary thyroid carcinoma, as explained by our biochemical finding of a high iodine content in the fluid. The pleural fluid thyroglobulin was also the highest reported to date.

## Case presentation

A 71-year-old Caucasian man diagnosed with metastatic papillary thyroid cancer was referred to the Pleural Clinic for management of a large right-sided effusion. He was diagnosed with papillary thyroid cancer (PTC) 11 years prior, and was treated with total thyroidectomy and adjuvant radio-iodine treatment. Mutation analysis was not performed. Over the next four to six years of follow-up, he received further radioactive iodine therapy due to disease recurrence with rising serum thyroglobulin levels. Small pulmonary metastases were first identified as fluorodeoxyglucose (FDG)-avid lesions on positron emission tomography-computed tomography (PET-CT) five years before this presentation, together with right-sided pleural thickening. Since then serial serum thyroglobulin showed continual rises from 51 to 1100ug/L over four years. The patient had iodine-refractory disease and was enrolled in a clinical trial of pazopanib, a multi-targeted receptor tyrosine kinase inhibitor, two years prior to his presentation to the Pleural Clinic. Treatment with pazopanib, significantly reduced his thyroglobulin level to 290ug/L.

He was an ex-smoker, with a history of ischaemic heart disease, peripheral vascular disease and asbestos exposure.

**Figure 1:**
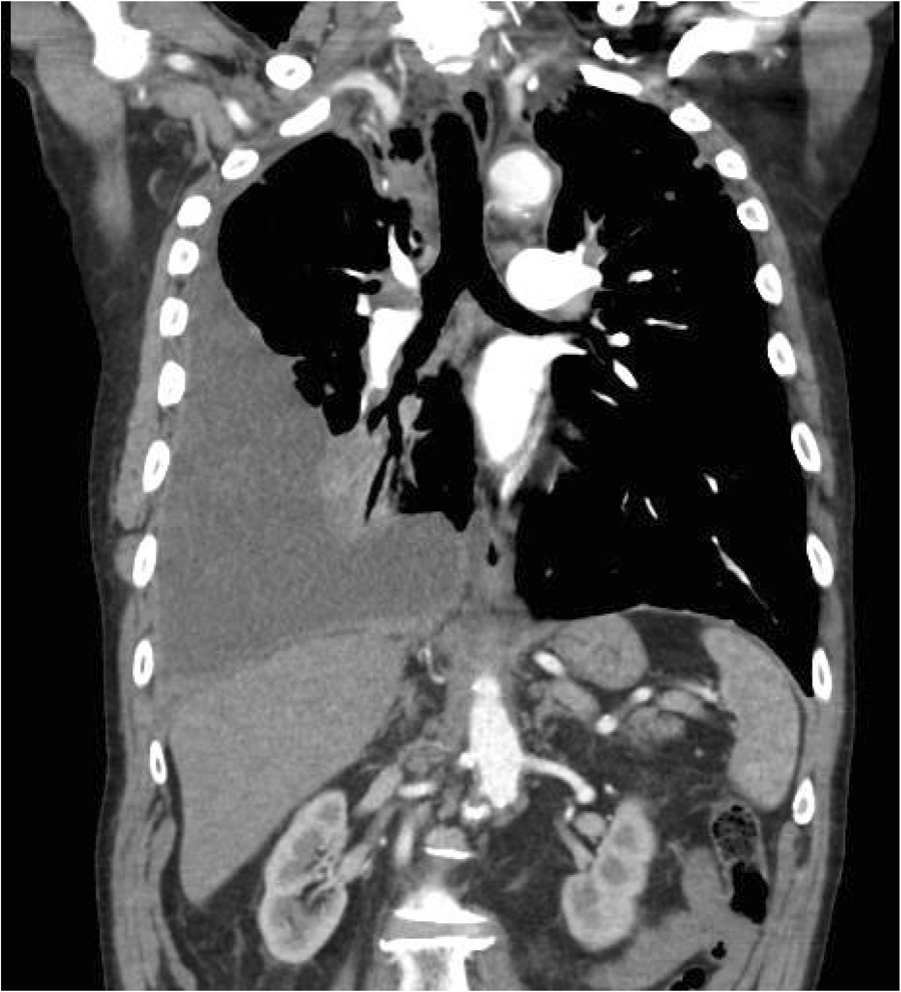
Chest computed tomography scan confirming a large, right-sided pleural effusion in the setting of metastatic papillary thyroid carcinoma, prior to drainage and pleuroscopy.

**Figure 2:**
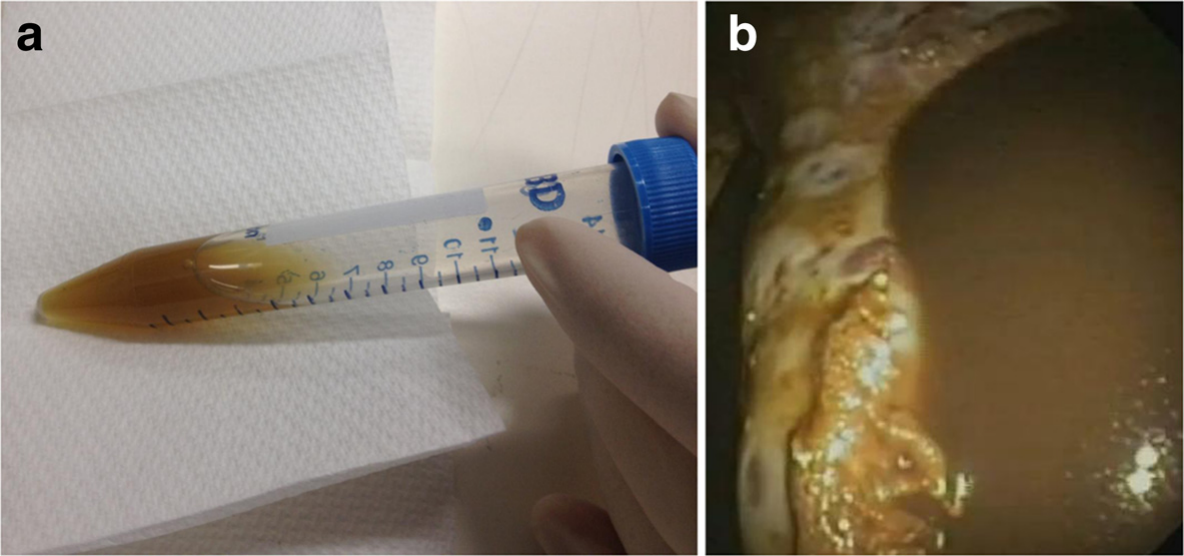
**Distinctive dark brown pigmentation of pleural fluid during pleuroscopy and persisting post fluid centrifugation. (a)** Pleural fluid with a distinctive dark brown pigmentation visible during pleuroscopy of the patient with metastatic papillary thyroid carcinoma. The lung remained trapped post pleuroscopy and drainage. **(b)** Persistence of brown pigmentation of pleural fluid post centrifugation, suggesting the presence of a dissolved substance later confirmed to be iodine.

When assessed at our Pleural Clinic, he was dyspnoeic and had a large, right-sided pleural effusion on CT scan (Figure 1) and ultrasound. A thoracentesis and a subsequent medical thoracoscopy both revealed an unusual dark brown pleural fluid (very similar in colour to commonly used iodine-based antiseptic solutions) (Figure 2a), also seen on the additional movie file (see Additional file 1), which remained after centrifugation (Figure 2b). The fluid was an exudate by Light’s criteria (protein 85g/L, lactate dehydrogenase (LDH) 712U/L) and had a pH of 7.13 and glucose 0.8mmol/L. Cytological and immunohistological features were consistent with adenocarcinoma, with positive thyroid transcription factor-1 (TTF-1) and PAX8 suggesting metastatic thyroid carcinoma. However, thyroglobulin staining was negative, making an unequivocal diagnosis difficult.

Medical thoracoscopy revealed multiple areas of tumour deposits on the parietal and visceral pleura, clearly shown in the additional movie file (see Additional file 1). Many of the tumour nodules had the same unique dark brown pigmentation as the pleural fluid. Histopathology showed papillary structures, colloid material, psamomma bodies and adenocarcinoma cells positive for TTF-1 and thyroglobulin, confirming a metastatic PTC.

Pleural fluid thyroglobulin was more than 80-fold higher than in corresponding serum (29,000 versus 350ug/L), and is the highest level yet reported. Given the unusual colour of the fluid and its striking resemblance to iodine solutions, we analysed the pleural fluid total iodine content using inductively coupled plasma mass spectrometry [10] and showed a high concentration of 23,000ug/L. Both the pleural fluid iodine and thyroglobulin concentrations were from the extracellular component of the fluid analysed.

The patient received a talc poudrage pleurodesis and continued with pazopanib therapy.

## Discussion

We report a case of metastatic PTC to the pleura and describe for the first time a high iodine content of the fluid that explains an unusual dark brown colour of the pleural fluid not previously reported.

Papillary thyroid cancer is the most common form of thyroid malignancy and metastatic disease tends to involve local lymph nodes [11]. Distant metastases to the lungs are uncommon; metastasis to the pleura is very rare. In one series, only 0.6% of PTC patients developed a malignant pleural effusion [4].

Inspection of the pleural fluid is often considered the first step in its analysis, and can provide clues to empyema, chylous effusions, haemothorax or oesophageal perforation. This is, however, the first report of a distinctive dark brown colour in a pleural effusion with a corresponding high iodine content in the fluid, presumably from synthesis of iodine-rich molecules by pleural PTC deposits. Brown effusions in themselves are not unique or pathognomic of metastatic PTC, and brownish effusions, of different color intensity, can be seen with degradation of haemoglobin, rupture of amoebic liver abscesses and parasitic infection [12]. Similar coloured fluid has been described in thyroid cysts but its iodine content was not measured [13]. How often a high iodine content and a brown effusion are present in PTC is unknown. However, the iodine-like colour of pleural fluid may alert clinicians to the possibility of metastatic PTC in the investigation of pleural effusions.

Serum thyroglobulin is well established as a monitoring method for disease activity in PTC [14]. In this setting, the patient’s significantly raised pleural thyroglobulin levels (almost two logs higher than in corresponding serum) are noteworthy, and this finding has been suggested as a potential adjunct diagnostic marker [5]. Indeed, the pleural fluid thyroglobulin concentration in our patient was the highest ever documented. This may reflect the extent of pleural tumour and/or the chronicity of the effusion. Our observation supports the suggestion of pleural fluid thyroglobulin (or its ratio over serum) as a potential biomarker for diagnosis, and it may reflect disease burden - though these suggestions require formal testing.

## Conclusions

This case is the first to report high iodine content and an associated distinctive brown colour in the malignant effusion fluid from metastatic PTC. The usefulness of these findings in aiding diagnosis of metastatic pleural disease from PTC deserves investigation. In the meantime, a dark brown iodine-coloured effusion should raise the consideration of metastatic thyroid diseases.

## Consent

Written informed consent was obtained from the patient for publication of this case report and any accompanying images. A copy of the written consent is available for review by the Editor-in-Chief of this journal.

## Competing interests

The authors declare that they have no competing interests.

## Authors’ contributions

AR analysed and interpreted the patient data regarding the pleural disease and fluid characteristics, reviewed the literature, and provided a major contribution to the writing of the manuscript. EML provided clinical and technical advice and performed the pleural fluid chemical analysis. MM provided clinical advice and reviewed the manuscript. YCGL performed the pleursocopy, edited the manuscript and advised on all aspects of the case report. All authors read and approved the final manuscript.

## Supplementary Material

Additional file 1 **Right pleural space during medical pleuroscopy.** The distinctive pleural fluid colour is demonstrated and lesions confirmed as metastatic papillary thyroid carcinoma shown on the parietal and visceral pleura. Click here for file
